# Pancolitis due to Tuberculosis Masquerading as Inflammatory Bowel Disease in a 43‐Year‐Old Woman: Diagnostic Challenges in a Resource‐Limited Setting—A Case Study and Literature Review From Ethiopia

**DOI:** 10.1155/crgm/9525315

**Published:** 2026-11-01

**Authors:** Alemayehu Abebe Guji, Gashahun Mekonnen Dissassa, Melese Abere Gizaw, Daniel Rebuma Bekele, Merid Lemma Kebede, Kedir Negesso Tukeni

**Affiliations:** ^1^ Department of Internal Medicine Jimma University Jimma Oromia Ethiopia ju.edu.et; ^2^ Department of Internal Medicine Division of Gastroenterology & Hepatology Jimma University Jimma Oromia Ethiopia ju.edu.et; ^3^ Department of Pathology Jimma University Jimma Oromia Ethiopia ju.edu.et; ^4^ Department of Clinical Radiology Jimma University Jimma Oromia Ethiopia ju.edu.et

**Keywords:** colonic malignancy, colonic tuberculosis, Crohn’s disease, Ethiopia, IBD, Jimma

## Abstract

**Background:**

Gastrointestinal tuberculosis (GI TB) predominantly affects the ileocecal region, making primary colonic involvement exceptionally rare. In TB‐endemic, resource‐limited settings, differentiating colonic TB from inflammatory bowel disease (IBD)—particularly Crohn’s disease (CD)—presents a formidable diagnostic dilemma owing to overlapping clinical, radiological, and ileocolonoscopic features, often leading to diagnostic delays, misdiagnosis, and subsequent complications.

**Initial Clinical Presentation, Treatment, and Diagnostic Re‐Evaluation:**

In May 2026, a 43‐year‐old Ethiopian woman presented to Jimma University Medical Center with a 10‐month history of progressive colicky abdominal pain, chronic watery diarrhea, postprandial nonbilious vomiting, and significant weight loss. Prior empirical regimens of antihelminthic agents and broad‐spectrum antibiotics administered at peripheral health facilities yielded no clinical improvement. Initial laboratory workup revealed microcytic hypochromic anemia (hemoglobin: 8.5 g/dL), a significantly elevated erythrocyte sedimentation rate (ESR) (92 mm/h), hypoalbuminemia (2.8 g/dL), and a normal carcinoembryonic antigen (CEA) level (4 ng/mL). Ileocolonoscopy demonstrated superficial ulceration involving the ascending colon and hepatic flexure in a continuous form with numerous inflammatory polyps, and a small deep ulceration at the terminal ileum. Initial histopathology demonstrated chronic active inflammation without evidence of malignancy or granuloma formation. With a presumed diagnosis of CD, treatment was initiated with prednisolone and azathioprine, which might have contributed to the suspected deterioration of the clinical condition, warranting re‐evaluation with contrast‐enhanced CT scan of the abdomen and pelvis, which demonstrated diffuse, long‐segment circumferential wall thickening involving the terminal ileum, the entire colon, and the rectum, alongside pericolic fat stranding, a mesenteric phlegmon, and multiple necrotic mesenteric lymph nodes, with the largest measuring 2 × 3 cm with peripheral rim enhancement. Fine‐needle aspiration cytology (FNAC) of the necrotic lymph nodes revealed epithelioid granulomas and characteristic caseous necrosis. With the above evidence, terminal ileitis and pancolitis due to TB were considered, immunosuppressive medications were discontinued, and standard antituberculosis therapy (2RHZE/4RH) was promptly initiated. The patient started to experience clinical improvement, achieving full resolution of her constitutional and abdominal symptoms and gaining weight at 1 month of follow‐up.

**Conclusion:**

Diagnosing GI TB—especially in atypical sites such as the colon—presents a critical challenge in resource‐limited, TB‐endemic settings, where differentiating it from IBDs, especially CD, is vital to prevent severe complications from inappropriate therapy. To avoid misdiagnosing GI TB as IBD (which risks disseminated infection from immunosuppression) or IBD as TB (which worsens bowel stricture, fistulae, and perforation), clinicians must perform mandatory pretreatment TB screening using radiologic, immunologic, molecular, and culture modalities before initiating immunosuppressive agents. Furthermore, diagnostic accuracy requires obtaining deep submucosal biopsies during ileocolonoscopy to detect characteristic granulomatous lesions if present. Finally, when an empirical anti‐TB trial is necessary, it must be strictly time‐limited and followed by objective re‐evaluation at 8 weeks—including repeat endoscopy and biopsy—rather than relying solely on clinical symptomatic improvement.

## 1. Introduction

Tuberculosis (TB) is one of the most common infectious diseases, contributing to significant mortality and morbidity [1, 2]. Although pulmonary TB is the most common form, extrapulmonary TB accounts for one‐third of cases, with lymph node, gastrointestinal (GI), pleural, musculoskeletal, central nervous system, and laryngeal TB being the most common [3, 4]. The challenge of diagnosing and managing GI TB, especially with uncommon site involvement such as colonic TB in low‐resource settings, is due to the lack of appropriate investigation modalities of choice (computerized tomography [CT], ileocolonoscopy, biopsy, culture, immunologic, and molecular diagnostic methods) to differentiate it from common masqueraders such as colonic malignancy and inflammatory bowel disease (IBD). In addition, the lack of routine availability of experts in the field may contribute to such delays [5–11]. Therefore, we believe that discussing a case of TB pancolitis with terminal ileitis that was initially misdiagnosed as Crohn’s disease (CD) in a low‐resource setting will add scientific and clinical value. Discussing this atypical presentation, its clinical management, and the diagnostic challenges inherent to low‐resource settings can assist practitioners in TB‐endemic regions in distinguishing uncommon manifestations of colonic TB from other differentials. This discernment enhances early diagnosis, optimizes patient management, and improves clinical outcomes while averting futile interventions such as inappropriate immunosuppressive therapy and radical surgical procedures.

### 1.1. Clinical Timeline

#### 1.1.1. Initial Presentation and Baseline Investigations

Clinical presentation: A 43‐year‐old woman from Ethiopia presented in May 2026 with a 10‐month history of diffuse colicky abdominal pain, intermittent watery diarrhea, significant weight loss (> 15 kg), intermittent vomiting of ingested food, and loss of appetite. She visited different primary care settings and was given antihelminths and empirical antibiotics, but there was no improvement. She had no melena, hematochezia, cough, prior TB history or contact, chronic medical conditions, or history of immunosuppressive drug use.

Physical examination: The patient was emaciated with a body mass index (BMI) of 15.5 kg/m^2^, blood pressure of 100/60 mmHg, pulse rate of 98 bpm, pale conjunctiva, nonicteric sclera, and normal oxygen saturation. Abdominal examination showed a protuberant abdomen, diffuse tenderness, and no palpable organomegaly. Digital rectal examination was unremarkable.

### 1.2. Baseline Investigations

Hematology and chemistry: Moderate microcytic hypochromic anemia (Hgb = 8.5 g/dL) with reduced MCV of 77 fL, and a peripheral smear showing hypochromic red cells with anisopoikilocytosis. Erythrocyte sedimentation rate (ESR) was elevated at 92 mm/h, and the serum albumin level was 2.8 g/dL, indicating hypoalbuminemia.

Stool examination and endoscopy: Stool examination revealed mucoid stool with no ova or parasites and a positive fecal occult test. Abdominopelvic ultrasound showed hepatic flexure thickening (∼7 cm long), but no organomegaly or peritoneal thickening. Upper GI endoscopy was unremarkable. Initial ileocolonoscopy showed a small deep mucosal ulcer at the terminal ileum with a long circumferential superficial ulcer with numerous inflammatory polyps at the hepatic flexure, extending to the mid‐ascending colon (Figure 1). Biopsy indicated chronic active inflammation with no atypical or malignant cells and no granuloma formation suggestive of TB. Serum carcinoembryonic antigen (CEA) was 4 ng/mL (normal).

**FIGURE 1:**
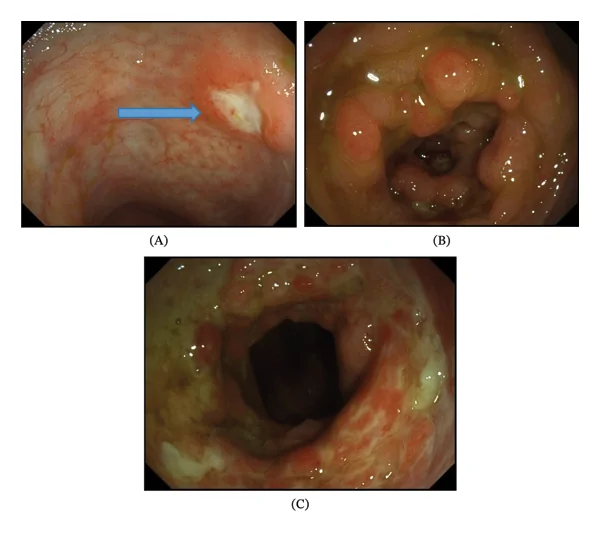
Findings from ileocolonoscopy. (A) A small, deep ulceration in the mucosa, measuring 0.5 cm by 0.5 cm, was observed in the terminal ileum (blue arrow). (B) The colonic mucosa contained numerous inflammatory polyps. (C) A longitudinal ulceration, circumferential in nature, extended from the hepatic flexure to the mid‐ascending colon, with no apparent luminal narrowing or blockage. The cecum, ileocecal valve, other colonic segments, and rectum exhibited normal mucosal architecture (not depicted). Multiple biopsy samples were taken from the lesions in both the ileum and colon in a separate container.

#### 1.2.1. Initial Differential Diagnosis and Rationale for CD

Colonic malignancy: Initially considered as a differential diagnosis due to the long duration of illness, presence of chronic refractory diarrhea despite antihelminthes and antibiotics, weight loss, night sweats, and ultrasound findings of a thickened hepatic flexure. Nevertheless, this possibility was dismissed as the CEA level was normal (4 ng/mL) and biopsy findings revealed only chronic active inflammation without any signs of malignancy or atypical cells.

Infectious causes (bacterial, protozoal, and parasitic): Deemed improbable because the patient did not exhibit immunosuppression or diseases such as HIV or diabetes, the prolonged illness did not align with acute bacterial causes, and stool tests consistently showed no abnormalities other than the mucoid nature and positive fecal occult. In addition, there was no response to the prior antibiotic and anthelmintic therapies.

CD: Provisional diagnosis of CD was made considering the clinical symptoms, endoscopic observations, and histopathological findings. The histopathology sample from the terminal ileum shows mucosal ulceration, erosion, and dense infiltration of the lamina propria with lymphoplasmacytic cells, while the colonic sample tissue revealed mucosal ulceration with significant infiltration of the lamina propria by lymphocytes, plasma cells, and fibrinopurulent exudates; caseating granulomas, which are typical of TB histopathology, were absent (Figure 2).

**FIGURE 2:**
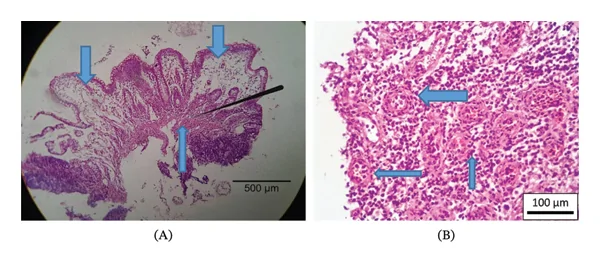
Hematoxylin and eosin staining: (A) terminal ileum: the photomicrograph of terminal ileal tissue sections reveals mucosal fragments bordered by unremarkable columnar epithelium, featuring areas of mucodepletion (thick arrows), reactive nuclear atypia, and surface erosion. Within the lamina propria, there is a dense infiltration of chronic lymphoplasmacytic inflammatory cells (thin arrow). (B) Colon: photomicrograph of sections from the colonic mucosa showing mucosal ulceration with significant infiltration of the lamina propria by lymphocytes, plasma cells, and fibrinopurulent exudates (thin arrows). The remaining crypts exhibit shortening and architectural distortion. Diffuse sheets of neutrophils and prominent vascular proliferation are also observed (thick arrow). Notably, there is no caseous necrosis or multinucleated giant cells present to suggest granuloma formation.

#### 1.2.2. Initiation of Prednisolone and Azathioprine

The patient commenced treatment with prednisolone at 40 mg and azathioprine 25 mg orally per day, along with supplemental multivitamins and oral iron, which were introduced 2 weeks following the initiation of treatment.

#### 1.2.3. Subsequent Clinical Course

One month following the commencement of treatment for CD, the patient exhibited no clinical improvement. Her abdominal pain, diarrhea, and constitutional symptoms persisted, and she did not gain weight.

#### 1.2.4. Diagnostic Re‐Evaluation and CT Findings

A contrast‐enhanced CT scan of the abdomen and pelvis was conducted for re‐evaluation. The scan revealed diffuse, long‐segment circumferential wall thickening affecting the terminal ileum, the entire colon, and the rectum. In addition, there was evidence of pericolic fat stranding, a mesenteric phlegmon, and multiple necrotic mesenteric lymph nodes, the largest measuring 2 cm by 3 cm with peripheral rim enhancement. These findings confirmed the possible progression of the disease from localized terminal ileum and limited colonic segment to pancolitis, which might be exacerbated due to immunosuppression (Figure 3).

**FIGURE 3:**
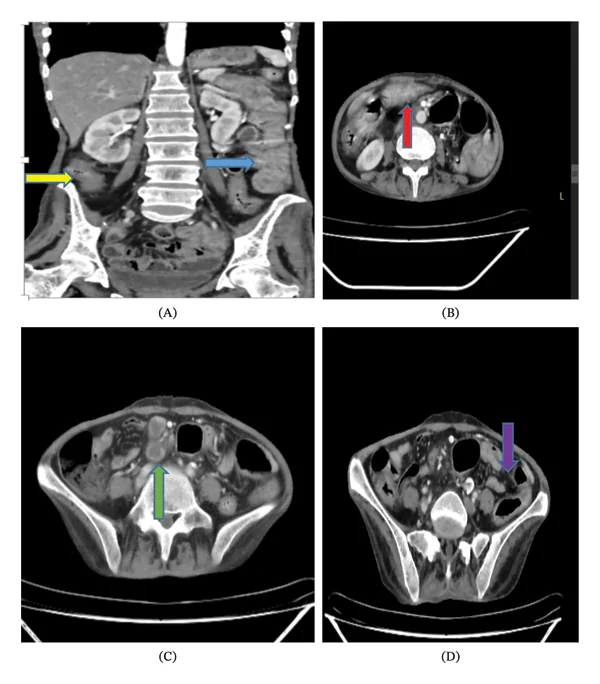
Contrast‐enhanced abdominal and pelvic computed tomography (CT) demonstrating: (A) wall thickening of the terminal ileum (yellow arrow) and descending colon (blue arrow), (B) wall thickening of the transverse colon (red arrow), (C) rim‐enhancing mesenteric lymphadenopathy with central necrosis (green arrow), and (D) rectal wall thickening (purple arrow). Associated pericolic fat stranding and a mesenteric phlegmon were also present (not shown).

#### 1.2.5. Lymph‐Node Sampling and Revised Diagnosis

Lymph‐node sampling: Ultrasound‐guided fine‐needle aspiration cytology (FNAC) of the necrotic mesenteric lymph nodes revealed epithelioid cell granulomas with characteristic caseous necrosis, providing strong evidence of TB (Figure 4).

**FIGURE 4:**
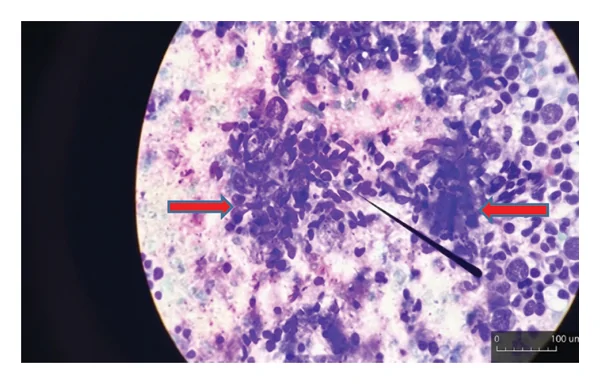
Fine‐needle aspiration (FNA) of a mesenteric lymph node (Giemsa stain). The photomicrograph demonstrates extensive amorphous granular necrotic debris, poorly formed (abortive) granulomas, and sheets of degenerated mixed inflammatory cells against a dirty background. Epithelioid granulomas with characteristic caseous necrosis are indicated by red arrows.

#### 1.2.6. Revised Diagnosis

The diagnosis was updated to pancolitis with terminal ileitis due to TB, as indicated by the lack of clinical improvement with immunosuppressive treatments, which was further supported by the suspected deterioration in the setting of immunosuppression, evidenced by imaging results that compared the initial abdominal ultrasound with the subsequent CT scans, which revealed mesenteric phlegmon, large necrotic mesenteric lymph nodes with rim enhancement, and involvement of the entire colon and rectum, findings not present in the initial ileocolonoscopy, and the histopathology sample from the mesenteric node, which suggested caseous necrosis with epithelioid granulomas. Because they were not available in our setup, we did not perform interferon‐gamma release assays (IGRAs), tuberculin skin testing (TST), or adenosine deaminase (ADA) activity assays during the initial or subsequent evaluations, despite their potential diagnostic value in this clinical scenario. Although a chest radiograph was not obtained during the baseline assessment, follow‐up chest radiography revealed no abnormalities.

#### 1.2.7. Anti‐TB Therapy

Following the updated diagnosis of tuberculous ileopancolitis, immunosuppressive therapy was immediately discontinued, and a standard anti‐TB regimen (2RHZE/4RH) was initiated. Iron supplementation was subsequently initiated once initial clinical stabilization was achieved.

#### 1.2.8. Clinical Response and Follow‐Up

Within 2 weeks of initiating anti‐TB therapy, the patient exhibited marked symptomatic improvement, with complete resolution of fever, diarrhea, abdominal pain, and night sweats, accompanied by early weight gain. At the 1‐month follow‐up assessment, her symptoms continued to resolve alongside a progressive increase in BMI. Hemodynamic parameters remained stable throughout the treatment course without requiring blood transfusion. The patient was maintained on scheduled clinical re‐evaluations, with repeat imaging and colonoscopy planned as clinically indicated.

## 2. Discussion

While GI TB is a well‐documented entity, presentation as ileopancolitis—as demonstrated in this index case—represents an uncommon manifestation that creates a substantial diagnostic challenge, particularly in resource‐limited settings with restricted diagnostic capabilities. Initially, the patient’s clinical presentation was attributed to infectious enterocolitis and CD, leading to empirical courses of antibiotics, antihelminthics, and immunosuppressive therapy across multiple facilities before TB was considered. The diagnosis was updated to pancolitis with terminal ileitis due to TB, as indicated by the lack of clinical improvement with immunosuppressive treatments, which was further supported by the potential deterioration in imaging findings and the histopathology sample from the mesenteric nodes. Due to resource constraints, IGRA, TST, and ADA activity assays were not performed during the initial or subsequent evaluations, despite their potential diagnostic value in this clinical scenario.

Differentiating GI TB from IBD remains particularly challenging in TB‐endemic regions, primarily due to two opposing diagnostic pitfalls. First, an empirical diagnosis of GI TB can lead to prolonged, unnecessary anti‐TB therapy in patients who actually have IBD. This treatment delay allows underlying IBD to progress, significantly increasing the risk of severe complications—such as stricture formation, bowel perforation, enterocutaneous fistulae, and poor long‐term outcomes [12, 13]. Conversely, misdiagnosing GI TB as IBD and initiating immunosuppressive therapy can trigger disseminated infection, disease exacerbation, and adverse patient outcomes [12]. In the current study, even if there was no strong evidence for ileopancolitis due to GI TB during the initial evaluation, we agree that immunosuppressive therapy was initiated without thorough and adequate workups.

Consequently, establishing a diagnosis of GI TB in endemic regions necessitates a comprehensive diagnostic strategy incorporating immunological assays, such as IGRA, TST, and ADA activity [12, 13]. Diagnostic evaluation should also encompass molecular and microbiological modalities, including tissue‐based GeneXpert MTB/RIF assays, liquid culture systems, such as the Mycobacterial Growth Indicator Tube (MGIT), and standard mycobacterial cultures [6, 7, 9]. However, in resource‐limited settings, these modalities are rarely accessible outside of GeneXpert, creating a critical diagnostic bottleneck when attempting to differentiate colonic TB from IBD, especially CD.

Another challenge in the diagnosis of colonic TB is the depth of the biopsy, in which a superficial biopsy may miss typical granulomatous lesions of TB because GI TB originates in the intestinal lymphoid aggregates located predominantly in the submucosal tissue, obviating the need for a deep submucosal biopsy [8, 14], which is very helpful to differentiate IBD, especially CD, from colonic TB.

Several case reports have described colonic masses with or without concurrent ileal and cecal area involvement, which were finally diagnosed as colonic TB, most of which were diagnosed during the initial visit [15–17]. However, in our case, the patient visited different health facilities without a diagnosis due to a lack of appropriate workup, as well as misdiagnosis as CD, leading to unnecessary immunosuppressive therapy, which might have worsened the initial clinical outcome.

The clinical implications of these findings are significant. First, in TB‐endemic settings, intestinal TB should be adequately assessed before initiating immunosuppressive treatment for presumed IBD, especially CD, particularly when the endoscopic, radiological, or clinical findings are not typical enough to establish CD with confidence [18, 19]. Second, when intestinal TB cannot reasonably be excluded, and an empirical anti‐TB trial is chosen, the trial should be time‐limited and followed by objective reassessment, which includes clinical monitoring, TB markers, culture for TB, and repeated endoscopic evaluation followed by biopsy at 8 weeks rather than simply continuing treatment based on symptomatic improvement alone [12, 13]. Third, the role of deep submucosal biopsy in differentiating IBD from GI TB should be taken into consideration [8, 14]. Lastly, from a public health perspective, ensuring access to adequate GI TB diagnosis methods such as endoscopy, CT, and pathology and integrating molecular, bacteriologic, and TB marker workups have paramount importance [10, 11, 20].

## 3. Conclusion

Diagnosing GI TB, particularly in atypical sites such as the colon, presents a major challenge in resource‐limited, TB‐endemic settings. Differentiating GI TB from IBDs, especially CD, is critical to avoid a dangerous diagnostic dilemma: misdiagnosing GI TB as IBD risks severe infection reactivation due to immunosuppression, whereas misdiagnosing IBD as GI TB delays essential treatment and leads to severe complications.

### 3.1. Recommendations

To ensure an accurate diagnosis and optimize clinical outcomes, practice should center on the following four key principles:

Mandatory pretreatment screening*:* Thoroughly screen for TB using chest radiography, immunologic tests (IGRA, ADA, and TST), molecular markers (PCR and GeneXpert MTB RIF assay), and culture before initiating immunosuppressive therapy for presumed IBD.

Deep submucosal biopsies: Perform deep biopsies during ileocolonoscopy, as superficial sampling frequently misses characteristic submucosal granulomatous lesions.

Time‐limited empirical trials: If an empirical anti‐TB trial is initiated, its duration should be strictly capped, and objective re‐evaluation should be mandated, including repeat endoscopy and biopsy at 8 weeks, rather than relying solely on symptomatic improvement.

Diagnostic expansion*:* Enhance public health access to advanced imaging such as CT, pathology, immunologic, and molecular diagnostic methods for TB to eliminate diagnostic bottlenecks of GI TB diagnosis in low‐resource settings.

### 3.2. Limitations

A primary limitation of this study is that the patient was not followed up through the completion of anti‐TB therapy to confirm clinical cure, nor was a repeat colonoscopy performed before manuscript revision. During the initial visit, an abdominal CT scan was not carried out, and due to limitations in local resources, immunologic, molecular, and bacteriologic tests for TB were not feasible during either the initial or follow‐up visits.

## Author Contributions

Alemayehu Abebe Guji, Gashahun Mekonnen Dissassa, and Melese Abere Gizaw contributed to the conception, design, investigation, analysis, and drafting of the initial manuscript. Alemayehu Abebe Guji, Kedir Negesso Tukeni, Daniel Rebuma Bekele, and Merid Lemma Kebede participated in supervision, data analysis, editing, and curation. All authors reviewed and modified the article.

## Funding

No funding was received for the conduct of this research or preparation of the manuscript.

## Disclosure

All authors approved the final version of the manuscript.

## Ethics Statement

Formal ethical approval was not required for this case report. Patient confidentiality was strictly maintained, and all data were used solely for this publication without any identifying information.

## Consent

Written informed consent was obtained from the patient for the use of clinical data and images in this publication. All personal identifiers were removed. A copy of the written consent is available for review by the Editor‐in‐Chief of this journal.

## Conflicts of Interest

The authors declare no conflicts of interest.

## Data Availability

The data that support the findings of this study are available from the corresponding author upon reasonable request.
